# Lineage-matched Oropouche virus mRNA-LNP vaccines confer complete, cross-protective immunity in mice

**DOI:** 10.1128/mbio.03655-25

**Published:** 2026-01-14

**Authors:** Yumiko Yamada, Inho Cha, Soowon Kang, Wan-Shan Yang, Morgan Lewis, Chloe Chung, Woo-Jin Shin, Jun Bae Park, Nam-Hyuk Cho, Young-Ki Choi, Natasha L. Tilston, Jae U. Jung

**Affiliations:** 1 Department of Microbial Sciences in Health, Cleveland Clinic Research, Cleveland Clinic 2569, Cleveland, Ohio, USA; 2 Global Center for Pathogen Research and Human Health Research, Cleveland Clinic Research, Cleveland Clinic 2569, Cleveland, Ohio, USA; 3 Department of Molecular Biology and Microbiology, Case Western Reserve University School of Medicine 12304, Cleveland, Ohio, USA; 4 Department of Microbiology and Immunology, Seoul National University College of Medicine, Seoul, Republic of Korea; 5 Department of Biomedical Sciences, Seoul National University College of Medicine, Seoul, Republic of Korea; 6 Center for Study of Emerging and Re-emerging Viruses, Korea Virus Research Institute, Institute for Basic Science, Daejeon, Republic of Korea; 7 Department of Microbiology and Immunology, Indiana University School of Medicine 12250, Indianapolis, Indiana, USA; Tsinghua University, Beijing, China

**Keywords:** Oropouche virus, mRNA vaccine, orthobunyavirus, cross-protective immunity, antigenic matching

## Abstract

Oropouche virus (OROV) is an emerging orthobunyavirus responsible for recurrent and increasingly widespread outbreaks across South and Central America, including a recent epidemic that elevated global concern. In addition to its known neurotropic potential—manifesting as meningitis and encephalitis—OROV is now associated with vertical transmission and adverse pregnancy outcomes, underscoring its growing clinical and public health relevance. The rapid geographic expansion and extensive genetic diversification of circulating OROV lineages highlight the need for vaccine platforms that can be rapidly updated to match evolving strains. To address this challenge, we developed mRNA-lipid nanoparticle (mRNA-LNP) vaccines encoding OROV envelope glycoproteins from a historical prototype lineage (OROV BeAn19991; Brazil) and a recently circulating outbreak lineage (OROV AM0059; Brazil). Both immunogens were human codon-optimized, validated for robust expression, and evaluated in BALB/c and A129 mice. Vaccination elicited strong humoral and cellular responses, including high-titer OROV-specific IgG, broad cross-neutralization of prototype and contemporary strains, and potent CD4^+^ and CD8^+^ T cell immunity. Each vaccine conferred complete protection against lethal OROV challenge. Notably, the outbreak-lineage vaccine achieved full protection against both prototype and outbreak strains, whereas the prototype-based vaccine provided only partial cross-protection. These findings establish mRNA-LNP vaccines as a powerful and adaptable strategy for combating OROV. Incorporating contemporary, strain-matched antigens markedly enhances protective breadth and potency, positioning this platform for rapid deployment against ongoing outbreaks and the rising threat posed by related emerging orthobunyaviruses.

**IMPORTANCE:**

Oropouche virus (OROV) is a re-emerging orthobunyavirus that causes recurrent outbreaks across Central and South America. Although OROV infection is often described as a self-limited febrile illness, neurological complications and, more recently, fetal abnormalities and deaths have been reported, reflecting both the true clinical impact of OROV and improved surveillance. Despite this, no licensed vaccines or antivirals are available. Given the virus’s segmented genome and extensive genetic diversity, an effective countermeasure must be both cross-protective and rapidly updateable as new lineages emerge. Here, we show that mRNA-lipid nanoparticle (mRNA-LNP) vaccines encoding OROV envelope glycoproteins from distinct viral lineages elicit strong antibody and T cell responses and provide robust protection in mouse models, including sterilizing immunity against lethal challenge with both prototype and currently circulating strains. Our data indicate that incorporating contemporary antigenic sequences can enhance cross-strain protection, supporting the use of mRNA-LNP platforms as a rapid, adaptable solution for future OROV outbreaks and related emerging pathogens.

## INTRODUCTION

Oropouche virus (OROV) is a re-emerging arbovirus belonging to the genus *Orthobunyavirus* (family *Peribunyaviridae*), transmitted predominantly by *Culicoides paraensis* (biting midges) (1) and less frequently by mosquitoes such as *Culex quinquefasciatus* and *Aedes serratus* (2). Human OROV infection typically presents as an acute febrile illness with headache, myalgia, and arthralgia, but severe neurological disease, including meningitis and encephalitis, has long been documented (3). Recent outbreaks, however, have coincided with a sharp increase in reported neurological complications. Whether this surge reflects true changes in viral pathogenicity or simply the consequence of unprecedented case numbers and improved clinical recognition remains unclear. Regardless, the rising burden of neuroinvasive disease underscores the need to better understand contemporary OROV strains and to develop vaccine platforms capable of responding to shifting clinical patterns (3).

Since its first isolation in Trinidad and Tobago in 1955, OROV has caused over 30 outbreaks throughout South and Central America, infecting an estimated half a million people (4). Urbanization, changes in vector ecology, and climate-driven shifts in habitat range are expected to further expand OROV transmission. Importantly, the recent outbreaks have revealed a broader and more severe clinical spectrum, including significant neurological disease (5, 6), growing evidence of vertical transmission leading to fetal abnormalities and adverse pregnancy outcomes (6–8), and nine confirmed human deaths (6). These observations highlight that OROV poses a more substantial and multifaceted public health threat than previously appreciated.

Like other orthobunyaviruses, OROV has a tripartite, negative-sense RNA genome (L, M, and S segments) that facilitates reassortment (9). Genomic surveillance has revealed substantial diversity among circulating OROV lineages, including multiple reassortant viruses that differ markedly from the historical Brazilian prototype strain BeAn19991 (10–12). This genetic and antigenic variability raises concerns that prototype-based vaccines may provide suboptimal protection against contemporary or emerging OROV strains.

Despite OROV’s expanding geographic footprint and escalating clinical relevance, no licensed vaccine or virus-specific therapeutics exist. Traditional vaccine platforms, such as inactivated or live-attenuated viruses, require lengthy development and manufacturing timelines that are incompatible with rapid response during outbreaks or following the identification of new lineages (13). An ideal OROV vaccine must therefore combine robust immunogenicity and cross-strain protection with the flexibility to be rapidly reprogrammed as viral sequences evolve.

mRNA vaccines formulated in lipid nanoparticles (mRNA-LNPs) offer these advantages (14, 15). They can be designed directly from viral genomic data, enabling rapid development without the need for pathogen cultures (16). mRNA-LNP vaccines elicit strong humoral and cellular immune responses and have demonstrated efficacy against diverse viral targets (17, 18), including our previous studies on SARS-CoV-2 (19), Kaposi’s sarcoma-associated herpesvirus (20), and severe fever with thrombocytopenia syndrome virus (21). Such features make mRNA-LNPs well-suited for OROV, where reassortment and lineage diversification continually generate novel antigenic profiles.

In this study, we developed and evaluated human codon-optimized mRNA-LNP vaccines encoding OROV envelope glycoproteins from a historical prototype lineage and a contemporary circulating outbreak lineage. We compared their immunogenicity, neutralizing breadth, and protective efficacy in murine models using lethal challenge with a prototype and a currently circulating clinical isolate. Our results show that OROV mRNA-LNP vaccines elicit strong and broadly cross-protective immune responses, and that contemporary antigenic sequences substantially enhance coverage against the diverse strains now circulating. These findings position mRNA-LNPs as a powerful, rapid-response vaccine platform for controlling Oropouche fever outbreaks and addressing its escalating clinical concerns, including neuroinvasion and vertical transmission.

## RESULTS

### Comparative sequence and structural analysis of OROV glycoproteins and design of mRNA vaccine constructs

To evaluate antigenic differences critical for vaccine design, we compared the envelope glycoprotein complexes of the well-characterized prototype strain OROV BeAn19991 (Brazil, 1960) and OROV AM0059, a contemporary clinical isolate associated with the 2024 resurgence in Brazil that reflects the antigenic diversity of currently circulating OROV. Gn and Gc form a surface heterodimer; across orthobunyaviruses, Gc is the principal target of neutralizing antibodies and a key mediator of entry and fusion (22–24), whereas Gn mainly supports virion assembly and shields Gc epitopes. Accordingly, structural and sequence comparisons focused on Gc as the most relevant antigen for neutralization-based immunity.

**Fig 1:**
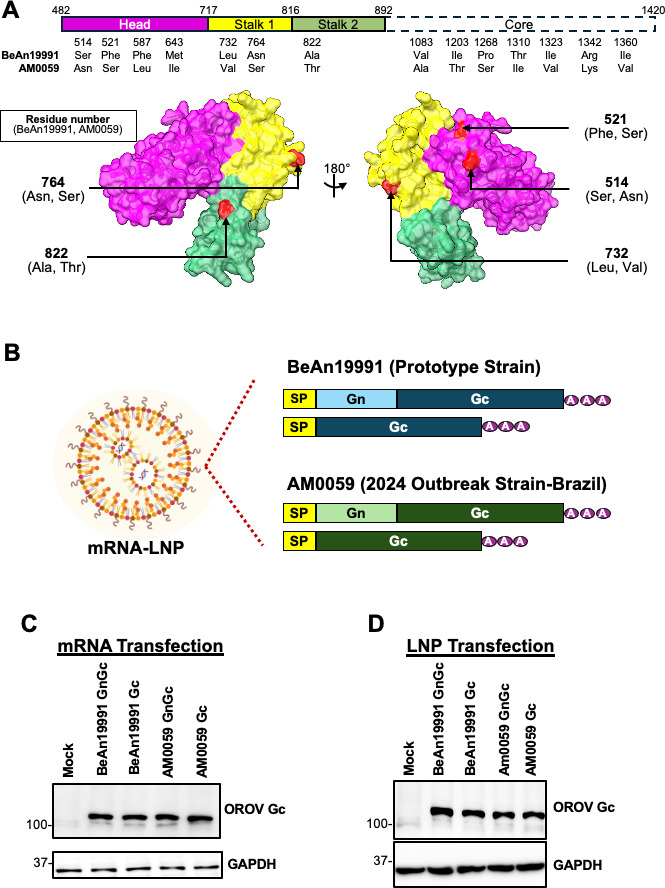
Comparative analysis of OROV glycoproteins and design of mRNA-LNP vaccine constructs. (**A**) Domain organization of the OROV Gc glycoprotein and structural comparison between the prototype BeAn19991 and outbreak AM0059 strains. Predicted surface models generated by AlphaFold3 highlight amino acid substitutions (red) distributed across the head (magenta), stalk 1 (yellow), stalk 2 (green), and core (white) domains. (**B**) Schematic representation of the LNP-encapsulated mRNA vaccine constructs encoding either the full-length Gn-Gc polyprotein or Gc alone from the BeAn19991 (blue) and AM0059 (green) strains. Each construct includes an N-terminal signal peptide (SP) and a 3′ poly(A) tail for transcript stabilization. Validation of OROV glycoprotein expression in HEK293T cells transfected with *in vitro*-transcribed (**C**) or LNP-formulated (**D**) mRNA. Protein lysates were collected 24 h post-transfection and analyzed by Western blot using anti-OROV Gc antibodies. GAPDH served as a loading control.

We compared Gc from the prototype BeAn19991 strain and the 2024 Brazilian isolate AM0059. Monomeric ectodomain structures were predicted with AlphaFold3 (25). Both models had high confidence (pTM = 0.86; Fig. S1A) and aligned well to Schmallenberg virus Gc (PDB: 6H3S [22]) (Fig. S1B). Sequence comparison revealed 14 amino acid substitutions—seven in the core domain and seven in the head/stalk region. Of the seven head/stalk substitutions, five are predicted to be surface-exposed (Fig. 1A). These substitutions did not produce global conformational changes or alter electrostatic surface potentials in a characteristic manner (Fig. S1B and C), suggesting that strain-specific modifications are more likely to modulate local dynamics or stability than to remodel the overall fold.

Although Gc is the dominant neutralizing antigen, we also engineered full-length GnGc constructs to test whether Gn affects antigen expression, folding, or immunogenicity *in vivo*. Based on the sequence analysis, we generated four human-codon-optimized mRNA-LNP constructs encoding either BeAn19991 or AM0059 glycoproteins: BeAn19991 GnGc, BeAn19991 Gc, AM0059 GnGc, and AM0059 Gc (Fig. 1B). Each transcript included an N-terminal signal peptide and a poly(A) tail; the NSm region, a non-structural membrane protein encoded within the M segment and not required for virion assembly or immunogenicity, was omitted (26).

HEK293T cells were transfected with plasmid DNA, and western blot results showed that only the human codon-optimized plasmids produced readily detectable levels of glycoprotein expression, highlighting the importance of codon optimization for efficient antigen production (Fig. S2A). We further tested using *in vitro*-transcribed mRNA (Fig. 1C) and LNP-formulated mRNA (Fig. 1D), and western blots with OROV Gc-specific antibodies demonstrated robust expression across all formats. To verify Gn expression, an additional GnGc construct bearing an N-terminal V5 tag was probed with anti-V5 antibody, confirming efficient co-expression of Gn and Gc at the expected sizes (Fig. S2B and C). Collectively, these data validate the vaccine design and demonstrate that both glycoproteins are produced as intended.

### Immunogenicity and neutralizing antibody responses elicited by OROV mRNA-LNP vaccines against prototype OROV strain (rOROV^BeAn19991^)

**Fig 2:**
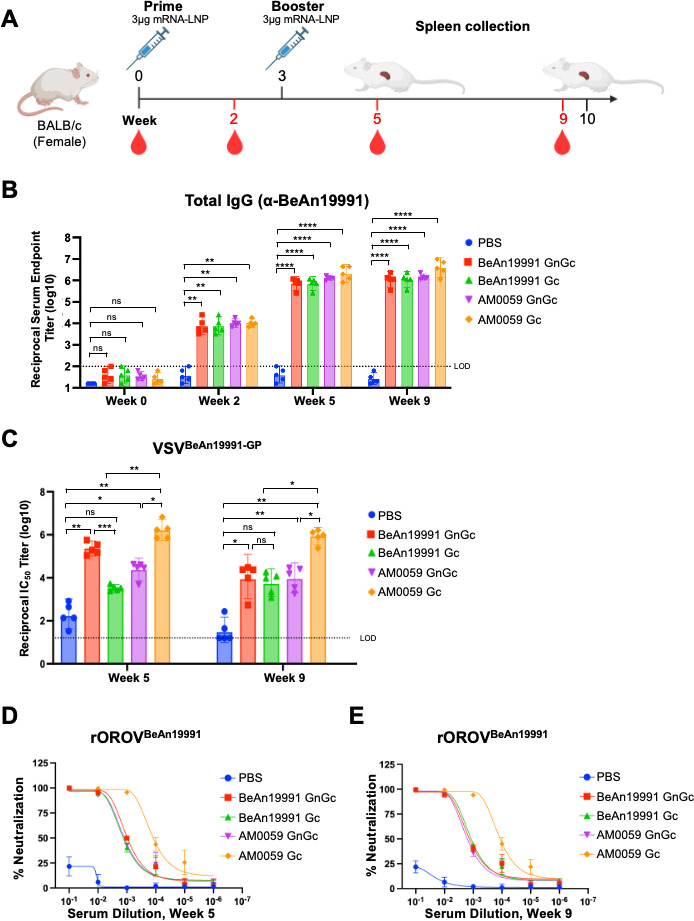
OROV mRNA-LNP vaccines elicit strong IgG and neutralizing antibody responses against the rOROV^BeAn19991^ strain. (**A**) Immunization and sample collection schedule in BALB/c mice (*n* = 5 per group). Mice were immunized intramuscularly at weeks 0 and 3 with LNP-formulated mRNAs encoding OROV glycoproteins from either the BeAn19991 or AM0059 strain, expressed as full-length Gn-Gc or Gc-only constructs. Sera were collected at weeks 0, 2, 5, and 9 for serological analysis. (**B**) Total anti-BeAn19991 IgG titers measured by ELISA against purified BeAn19991 virion lysate. The dashed line indicates the limit of detection. Statistical significance was assessed by one-way analysis of variance (ANOVA) with Tukey’s multiple comparisons test. No statistically significant differences were detected among the vaccinated groups. (**C**) Neutralization activity of sera collected at weeks 5 and 9 against VSV-based pseudoviruses displaying BeAn19991 glycoproteins (GP). (**D, E**) Validation of neutralizing activity using authentic rOROV^BeAn19991^ virus in a FRµNT with the same sera. Data represent mean ± SEM (*n* = 5). Statistical analysis was performed using one-way ANOVA followed by Tukey’s multiple comparisons test. Statistical significance was defined as follows: *P* ≤ 0.05 (*), *P* ≤ 0.01 (**), *P* ≤ 0.001 (***), and *P* ≤ 0.0001 (****), with “ns” for non-significant findings.

To characterize the antibody responses induced by the codon-optimized OROV mRNA-LNP vaccines, we immunized BALB/c mice intramuscularly with LNP-formulated mRNAs encoding either the BeAn19991 or AM0059 glycoprotein, expressed as full-length GnGc or Gc-only constructs. Mice received a prime-boost regimen at weeks 0 and 3, and sera were collected at weeks 0, 2, 5, and 9 (Fig. 2A).

Humoral responses were first assessed by ELISA using purified BeAn19991 virion lysate to quantify prototype strain-specific antibodies. All vaccinated groups developed significantly higher total anti-BeAn19991 IgG titers than the phosphate-buffered saline (PBS) group (Fig. 2B). IgG levels were markedly increased after the booster immunization, indicating a strong immune response. Although all mRNA-LNP formulations induced comparable antibody titers, no significant differences were observed between the GnGc- and Gc-only constructs, suggesting that both vaccine designs elicited similarly robust systemic IgG response against the BeAn19991.

Neutralizing capacity was next examined using a VSV-based pseudovirus displaying the BeAn19991 glycoprotein. All vaccine constructs elicited strong pseudovirus-neutralizing activity, as shown by high 50% inhibitory concentration (IC_50_) titers measured at weeks 5 and 9 following the booster immunization (Fig. 2C). Notably, the AM0059 Gc construct induced the highest neutralizing titers against the BeAn19991 pseudovirus, indicating that antibodies generated from Brazilian isolate AM0059 antigen effectively recognized and neutralized the prototype strain. In contrast, sera from PBS-treated mice showed no inhibition of pseudovirus entry, confirming the specificity of vaccine-induced responses.

To further confirm these findings, we evaluated neutralization using the authentic rOROV^BeAn19991^ strain in a focus reduction microneutralization test (FRµNT). Consistent with the pseudovirus results, sera from all vaccinated groups showed significantly higher neutralization activity than the PBS control (Fig. 2D and E), with corresponding infection data provided in Fig. S3A and B. Among the vaccine formulations, sera from the AM0059 Gc group exhibited the highest neutralization activity, significantly exceeding the other vaccine groups, whereas PBS sera showed no detectable inhibition.

Together, these results demonstrate that the human codon-optimized OROV mRNA-LNP vaccines elicit robust antibody responses directed against the prototype rOROV^BeAn19991^ strain, characterized by high systemic IgG levels and strong neutralizing activity in both pseudovirus and authentic-virus assays.

### Immunogenicity and neutralizing antibody responses elicited by OROV mRNA-LNP vaccines against the outbreak OROV strain (OROV^240023^)

**Fig 3:**
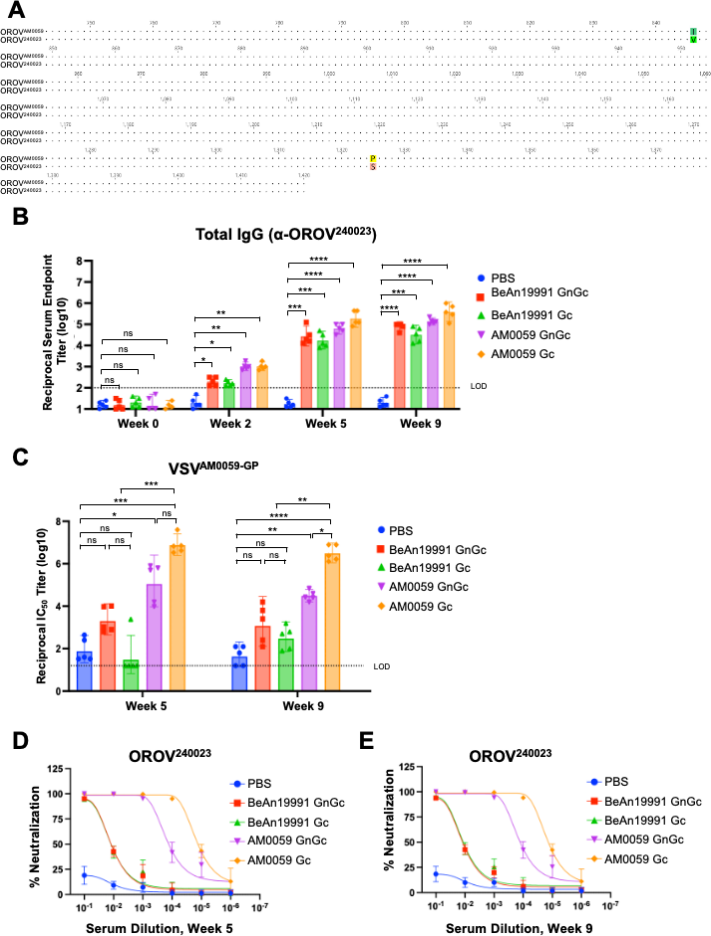
OROV mRNA-LNP vaccines elicit differential neutralizing antibody responses against the outbreak strain OROV^240023^. (**A**) Amino acid sequence alignment of OROV^AM0059^ and OROV^240023^ Gc glycoproteins highlighting the sites of sequence variation. (**B**) Total anti-OROV^240023^ IgG titers measured by ELISA using purified OROV^240023^ virion lysate. The dashed line indicates the limit of detection. Statistical significance was assessed by one-way ANOVA with Tukey’s multiple comparisons test. No statistically significant differences were detected among the vaccinated groups. (**C**) Neutralizing activity of sera collected at weeks 5 and 9 against VSV-based pseudoviruses displaying the AM0059 glycoproteins (GP). (**D, E**) Validation of neutralization using authentic OROV^240023^ virus in a FRµNT performed with the same sera. Data are presented as mean ± SEM (*n* = 5). Statistical analysis was performed using one-way ANOVA followed by Tukey’s multiple comparisons test. Statistical significance was defined as follows: *P* ≤ 0.05 (*), *P* ≤ 0.01 (**), *P* ≤ 0.001 (***), and *P* ≤ 0.0001 (****), with “ns” for non-significant findings.

Having established that both prototype- and outbreak-based mRNA vaccines elicit strong antibody responses against the prototype BeAn19991 strain, we next evaluated the breadth of these responses against a more recently isolated OROV strain. Because the Brazilian AM0059 isolate was unavailable for downstream assays, we used the closely related Cuba isolate OROV^240023^, obtained from BEI Resources (NIAID, NIH). This choice is epidemiologically relevant, as over 100 imported OROV cases linked to Cuba have been reported in the United States, underscoring the importance of assessing vaccine responses against this circulating lineage (6). Pairwise comparison of the OROV^AM0059^ and OROV^240023^ Gc glycoproteins revealed 99.79% sequence identity and 99.89% amino acid similarity, differing by only two residues (one conservative), indicating that OROV^240023^ serves as an appropriate surrogate for evaluating recognition of contemporary OROV variants (Fig. 3A). Sera from mice immunized with either BeAn19991- or AM0059-based constructs were analyzed for binding and neutralizing activity against OROV^240023^ antigens. The immunization regimen was identical to that used in the prototype strain study (Fig. 2A).

ELISA using purified OROV^240023^ virion lysate revealed a significant increase in total IgG titers across all vaccinated groups following prime-boost immunization (Fig. 3B). Antibody levels were comparable among the constructs, indicating that both BeAn19991- and AM0059-based vaccines effectively elicited binding antibodies against the outbreak antigen.

Functional neutralization was first evaluated using a VSV-based pseudovirus displaying the AM0059 glycoprotein. As expected, sera from AM0059-vaccinated mice exhibited strong, dose-dependent inhibition of pseudovirus entry at weeks 5 and 9, consistent with an antigen-matched response (Fig. 3C). In contrast, sera from BeAn19991-vaccinated mice showed minimal inhibition, suggesting weaker neutralization against the outbreak antigen.

To further validate these observations using authentic virus, we next performed FRµNT assays with the OROV^240023^ strain. Consistent with the pseudovirus results, sera from AM0059-vaccinated mice displayed significantly higher neutralizing titers than sera from BeAn19991-vaccinated mice (Fig. 3D and E), with corresponding infection data provided in Fig. S3C and D. The PBS control group showed no viral entry inhibition.

Collectively, these data confirm that AM0059 mRNA-LNP vaccination induces potent neutralizing antibodies that recognize both pseudotyped and authentic OROV^240023^ viruses. The reduced neutralization observed in BeAn19991-immunized sera occurred despite comparable binding titers. This finding suggests that even subtle amino acid changes in Gc can alter neutralization sensitivity and impact vaccine performance.

### B cell activation, cytokine secretion, and T cell responses induced by OROV mRNA-LNP vaccination

To characterize the immune responses elicited by OROV mRNA-LNP vaccines beyond neutralizing antibody production, we assessed antigen-specific B cell, cytokine, and T cell responses in vaccinated mice at weeks 5 and 10 post-immunization.

**Fig 4:**
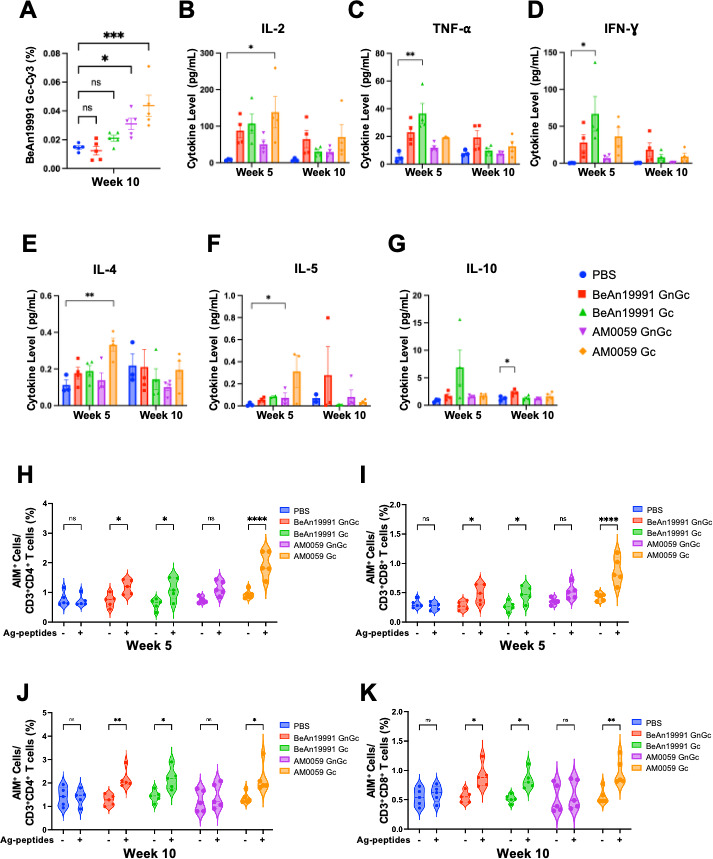
OROV mRNA-LNP vaccines elicit coordinated humoral and cellular immune responses. (**A**) Frequency of BeAn19991 Gc^+^ B cells in spleens of vaccinated mice at week 10, quantified by flow cytometry using Cy3-labeled recombinant BeAn19991 Gc antigen. (**B–G**) Serum cytokine measured by multiplex assay. Shown are representative concentrations of IL-2, IFN-γ, TNF-α, IL-4, IL-5, and IL-10. Data are presented as mean ± SEM. Statistical significance was evaluated using one-way ANOVA with Sidak’s *post hoc* correction for multiple comparisons. (**H–K**) Flow cytometric analysis of CD4^+^ and CD8^+^ T cell activation after *ex vivo* stimulation with BeAn19991 Gc overlapping peptides at week 5 and week 10. Data are presented as mean ± SEM. Statistical significance was determined by Student’s *t*-test. Statistical significance was defined as follows: *P* ≤ 0.05 (*), *P* ≤ 0.01 (**), *P* ≤ 0.001 (***), and *P* ≤ 0.0001 (****), with “ns” for non-significant findings.

First, BeAn19991 Gc-specific B cells were quantified in week 10 splenocytes by flow cytometry using a Cy3-conjugated recombinant BeAn19991 Gc probe (Fig. S4A). Vaccination increased the frequency of Gc^+^ B cells relative to PBS controls (Fig. 4A), with the AM0059 Gc group showing the highest proportion of antigen-specific B cells.

Next, we stimulated splenocytes with BeAn19991 Gc overlapping peptide pools and analyzed cytokine secretion to investigate vaccine-induced immune responses (Fig. 4B through G). Vaccinated mice exhibited elevated levels of Th1-associated cytokines, such as IL-2, IFN-γ, and TNF-α, and a moderate increase in Th2-associated cytokines, such as IL-4, IL-5, and IL-10, indicating a Th1-skewed immune response. Among the groups, AM0059-based vaccines appeared to induce higher overall cytokine concentrations, consistent with their stronger B cell activation profiles.

Finally, antigen-specific T cell responses were measured *ex vivo* after restimulation with the same peptide pools. Both CD4^+^ and CD8^+^ T cell activation increased in vaccinated mice compared with PBS controls (Fig. 4H through K). AM0059 Gc vaccination elicited the most pronounced activation of both subsets at weeks 5 and 10, including upon heterologous restimulation with BeAn19991 antigen, supporting enhanced cross-reactive cellular immunity.

Overall, these results show that OROV mRNA-LNP vaccines induce robust adaptive immunity characterized by OROV-specific B cells, a Th1-skewed cytokine profile, and strong CD4^+^/CD8^+^ T cell responses. The AM0059 Gc vaccine generated the most potent and broadly reactive cellular responses, consistent with its superior neutralizing activity.

### Protective efficacy of OROV mRNA-LNP vaccines against prototype rOROV^BeAn19991^ challenge in mice

To determine vaccine-mediated protection against OROV infection, the *in vivo* efficacy of the OROV mRNA-LNP vaccines was assessed in a lethal challenge model. Because GnGc and Gc-only formulations elicited comparable immunogenicity, subsequent studies focused on Gc constructs from the BeAn19991 and AM0059 strains.

**Fig 5:**
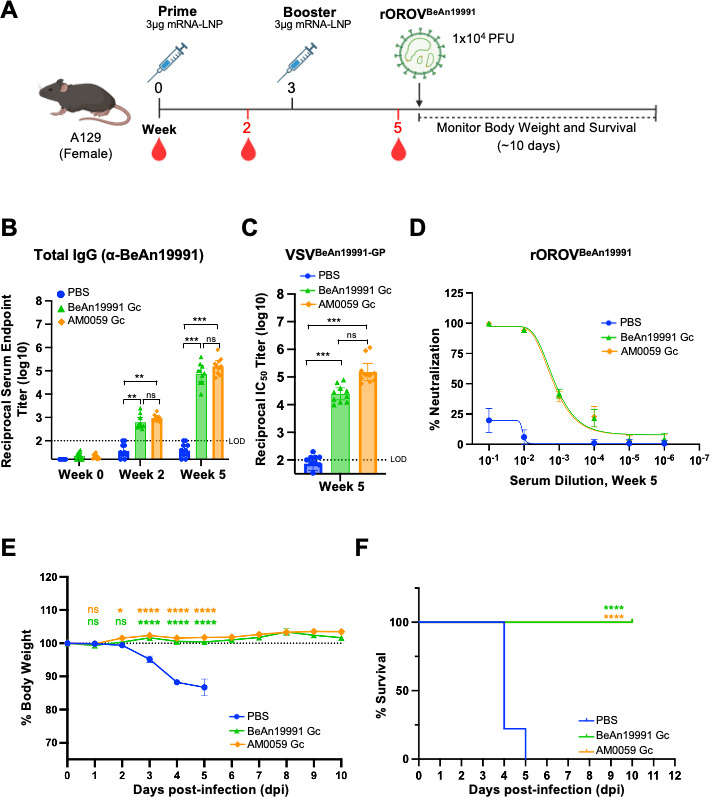
OROV mRNA-LNP vaccines confer complete protection against lethal rOROV^BeAn19991^ challenge in mice. (**A**) Experimental timeline of the vaccination and challenge protocol. A129 mice (PBS, *n* = 9; BeAn19991 Gc, *n* = 10; AM0059 Gc, *n* = 10) were immunized intramuscularly at weeks 0 and 3 with LNP-formulated mRNAs encoding Gc from either the BeAn19991 or AM0059 strain. At week 5, mice were challenged with a lethal dose (1 × 10^4^ PFU) of authentic rOROV^BeAn19991^ and monitored for 10 days post-infection. (**B**) Total anti-BeAn19991 IgG titers were quantified by ELISA using sera collected at weeks 0, 2, and 5. The dashed line indicates the limit of detection. Statistical significance was assessed by one-way ANOVA with Tukey’s multiple comparisons test. No statistically significant differences were detected among the vaccinated groups. (**C**) Neutralization activity of week-5 sera against VSV pseudoviruses expressing BeAn19991 glycoproteins. (**D**) Neutralization of rOROV^BeAn19991^ virus by FRµNT. Infected mice were monitored for (**E**) body-weight changes and (**F**) survival. Data represent mean ± SEM. Statistical analyses were performed using one-way ANOVA with Tukey’s *post hoc* test and log-rank (Mantel-Cox) test for survival. Statistical significance was defined as follows: *P* ≤ 0.05 (*), *P* ≤ 0.01 (**), *P* ≤ 0.001 (***), and *P* ≤ 0.0001 (****), with “ns” for non-significant findings.

A129 mice received intramuscular prime-boost at weeks 0 and 3 with LNP-encapsulated mRNA encoding either BeAn19991 Gc or AM0059 Gc. To evaluate antibody responses before viral challenge, sera were collected at weeks 2 and 5 for serological analysis (Fig. 5A). ELISA showed a marked increase in total anti-BeAn19991 IgG titers in both vaccinated groups. In contrast, PBS controls remained seronegative (Fig. 5B). Consistent with previous immunogenicity results, both vaccines induced potent neutralizing activity against the prototype OROV strain in VSV-based pseudovirus and authentic-virus FRµNT assays (Fig. 5C) with corresponding infection data provided in Fig. S3E. No significant difference was observed between the BeAn19991 Gc and AM0059 Gc formulations, indicating that both antigens elicited comparable pre-challenge neutralizing responses against the prototype virus.

At week 5, the mice were challenged subcutaneously via hock injection with a lethal dose (1 × 10^4^ plaque-forming units [PFU]) of authentic BeAn19991 virus, and clinical outcomes were monitored for 10 days. Both vaccinated groups maintained stable body weight, whereas PBS-treated mice exhibited rapid weight loss beginning on day 3 post-infection and succumbed by day 5 (Fig. 5E). Notably, all mice vaccinated with either BeAn19991 Gc or AM0059 Gc survived the challenge, whereas 100% of control mice died within 5 days (Fig. 5F).

Taken together, these data demonstrate that OROV mRNA-LNP vaccination provides complete protection against lethal rOROV^BeAn19991^ infections. The equivalent protection achieved by BeAn19991 Gc and AM0059 Gc constructs indicates that the Gc antigen alone is sufficient to elicit robust protective immunity against the prototype strain.

### Protective efficacy of OROV mRNA-LNP vaccines against the newly emerged outbreak strain OROV^240023^

**Fig 6:**
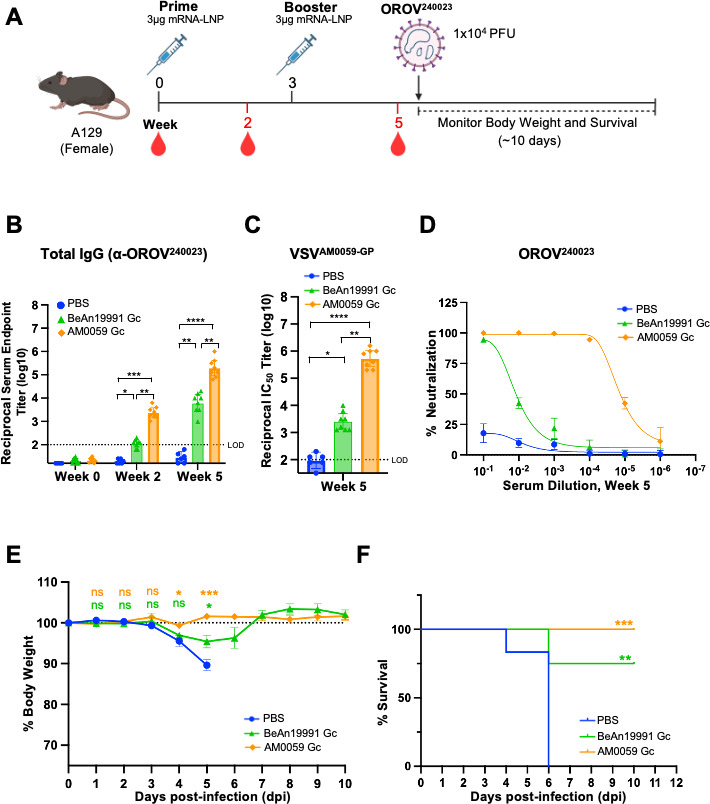
AM0059-based OROV mRNA-LNP vaccine provides complete protection against the newly emerged OROV^240023^ strain. (**A**) Schematic of the vaccination and challenge study. BALB/c mice (PBS, *n* = 6; BeAn19991 Gc, *n* = 8; AM0059 Gc, *n* = 8) were immunized with LNP-formulated BeAn19991 Gc or AM0059 Gc mRNAs at weeks 0 and 3, subsequently challenged with a lethal dose of authentic OROV^240023^ virus at week 5. (**B**) Total anti-OROV^240023^ IgG titers measured at week 5. The dashed line indicates the limit of detection. Statistical significance was assessed by one-way ANOVA with Tukey’s multiple comparisons test. No statistically significant differences were detected among the vaccinated groups. (**C, D**) Neutralization of pseudovirus expressing AM0059 glycoprotein (**C**) and authentic OROV^240023^ virus (**D**) by sera collected prior to challenge. (**E**) Body-weight changes and (**F**) survival following OROV^240023^. Data represent mean ± SEM. Statistical analyses were performed using one-way ANOVA with Tukey’s *post hoc* test and log-rank (Mantel-Cox) test for survival. Statistical significance was defined as follows: *P* ≤ 0.05 (*), *P* ≤ 0.01 (**), *P* ≤ 0.001 (***), and *P* ≤ 0.0001 (****), with “ns” for non-significant findings.

To determine whether the OROV mRNA-LNP vaccines protect against newly circulating strains, we evaluated their efficacy against the recent outbreak strain (Cuba isolate OROV^240023^) (Fig. 6A).

Prior to the viral challenge, both mRNA-LNP vaccine formulations elicited strong antibody responses, consistent with earlier immunogenicity findings. By week 5, sera from vaccinated mice exhibited high anti-OROV^240023^ IgG titers (Fig. 6B) and robust neutralizing activity against both pseudotyped VSV^AM0059-GP^ and authentic OROV^240023^ viruses, with the AM0059 Gc group showing the stronger response (Fig. 6C and D, corresponding infection data provided in Fig. S3F). These results confirmed that both vaccines remained immunogenic prior to the challenge, with the AM0059 Gc vaccine inducing stronger neutralization against the outbreak strain.

Following a lethal challenge with the authentic Cuba isolate OROV^240023^ virus, mice were monitored for 10 days for body weight and survival (Fig. 6E and F). The AM0059 Gc vaccine conferred complete protection, with 100% survival and stable body weights throughout the observation period. In contrast, BeAn19991-Gc-vaccinated mice exhibited partial protection (75% survival) accompanied by moderate weight loss, indicating reduced efficacy against the contemporary isolate. All PBS control mice succumbed to infection between 4 and 6 days post-challenge.

Together, these results demonstrate that both OROV mRNA vaccines are protective, but the AM0059-Gc formulation provides enhanced efficacy against the Cuba isolate OROV^240023^. This suggests incorporating contemporary sequence variants to improve cross-strain protection as OROV continues to evolve.

## DISCUSSION

In this study, we developed the first mRNA-LNP vaccines targeting OROV, addressing an urgent need for countermeasures against a re-emerging arbovirus responsible for recurrent outbreaks in South America. No licensed vaccines or therapeutics are currently available for OROV, despite its expanding range and rising incidence. Based on prior evidence that the Gc glycoprotein is the principal target of neutralizing antibodies and a key mediator of membrane fusion among orthobunyaviruses (22–24), we evaluated mRNA constructs encoding either Gc alone or the full-length GnGc precursor. Both designs produced robust antigen expression following plasmid DNA transfection, *in vitro* transcription, and LNP formulation (Fig. 1C and D; Fig. S2), confirming the effectiveness of human codon optimization and the mRNA-LNP platform. Only human codon-optimized constructs generated detectable protein expression in HEK293T cells, whereas constructs encoding wild-type viral codon sequences produced expression levels below the detection threshold. Orthobunyavirus glycoprotein genes exhibit pronounced codon bias, enrichment of AU-rich regions, and frequent usage of codons suboptimal for mammalian translation, which together likely reduce translational efficiency and mRNA stability in mammalian cells (27). Codon optimization appears to increase GC content, improve ribosomal processivity, and enhance protein expression, consistent with observations reported for other bunyavirus glycoproteins. We also observed slightly higher apparent protein levels in the V5-tagged GnGc constructs in Fig. S2C; this most likely reflects enhanced antibody recognition conferred by the small N-terminal V5 epitope rather than a true biological increase in expression. Similar modest improvements in detection have been reported for viral glycoproteins when V5 or HA tags improve immunodetection efficiency (28). Importantly, both tagged and untagged constructs supported efficient antigen production, validating the integrity of the vaccine design.

Comprehensive immune profiling revealed that OROV mRNA-LNP vaccination triggered a coordinated adaptive response engaging both humoral and cellular arms of immunity. Strong B cell activation was evidenced by the induction of Gc-specific B cells and high antigen-specific IgG titers. The accompanying cytokine profile, dominated by IFN-γ, TNF-α, and IL-2, with moderate levels of IL-4, IL-5, and IL-10, suggests a predominantly Th1-oriented immune response that favors antiviral protection. Such polarization supports class-switched antibody production, cytotoxic T cell priming, and long-term memory formation (29–32). Consistent with this, splenocytes from vaccinated mice exhibited activation of both CD4^+^ and CD8^+^ T cells after antigen restimulation, confirming that the vaccine elicited functional cellular immunity in addition to humoral protection. Similar findings have been reported for other mRNA vaccine platforms, where coordinated B and T cell activation enhances antibody maturation, cytotoxic activity, and durable protection (33–35). Among the formulations, AM0059 Gc induced the most coordinated B- and T cell activation, which may contribute to its enhanced neutralizing potency and protective efficacy observed *in vivo*.

Although our data indicate that both humoral and cellular immunity are robustly activated by OROV mRNA-LNP vaccination, the relative contributions of each arm to protection remain an important consideration for orthobunyaviruses. Neutralizing antibodies targeting Gc are likely the dominant correlate of protection, consistent with studies of related bunyaviruses such as Schmallenberg virus and Rift Valley fever virus, where Gc-specific antibodies prevent viral attachment and membrane fusion (36–38). However, the segmented genome of orthobunyaviruses enables reassortment events that may alter antigenic determinants on the Gc head and stalk domains (37, 39). In such scenarios, T cell-mediated immunity may provide a critical safeguard by eliminating infected cells and mitigating disease even when neutralization is partially compromised. Prior work in both bunyavirus and flavivirus systems demonstrates that vaccine-elicited CD4^+^ and CD8^+^ T cells can reduce viral burden and limit pathogenesis independently of antibodies (29–31, 40). The strong Th1-skewed response and potent CD4^+^/CD8^+^ activation induced by the AM0059 Gc vaccine suggest that mRNA platforms can generate cross-reactive cellular immunity that may preserve protection against drifted or reassortant OROV variants.

Challenge experiments provided direct evidence of vaccine-mediated protection. Because immunocompetent mice do not develop overt OROV disease, protective efficacy was evaluated in IFNAR1^−/−^ A129 mice, whereas BALB/c mice were used for immunogenicity analyses to preserve intact innate and adaptive immune signaling (1). Both BeAn19991 and AM0059 Gc formulations fully protected mice from lethal challenge with the prototype rOROV^BeAn19991^ strain. However, against the contemporary outbreak strain (Cuba isolate OROV^240023^), only the AM0059 Gc vaccine conferred complete survival, whereas BeAn19991 Gc offered partial protection. These results demonstrate that subtle antigenic differences within the Gc domain can affect neutralization sensitivity and *in vivo* efficacy. This finding is consistent with previous reports showing reduced cross-neutralization between prototype and outbreak OROV isolates (39). Comparable phenomena have been documented for Schmallenberg virus, where single substitutions in the Gc head domain generated escape from neutralizing mAbs and diminished susceptibility to polyclonal antisera (e.g., Y541C escape in Gc), and where M-segment variability (“hot-spot” in Gc) mediates immune escape (37). For phleboviruses such as Rift Valley fever virus, neutralizing activity maps to Gn/Gc, and protective mAbs target fusion/entry sites, reinforcing that epitope-localized changes can shift neutralization profiles (38). In summary, these observations support our conclusion that fine antigenic specificity within OROV Gc is a key determinant of cross-strain protection and argue for incorporating up-to-date outbreak sequences into vaccine design.

The enhanced protection conferred by the AM0059 Gc vaccine demonstrates the value of antigenic matching in vaccine development against rapidly evolving RNA viruses. This principle mirrors established strategies for influenza (41, 42) and SARS-CoV-2 (41, 43), where periodic updates sustain protection against drifted variants. Applying a similar, genomically informed framework to OROV and other orthobunyaviruses could mitigate immune escape as new lineages emerge. The adaptability of the mRNA-LNP platform makes this approach feasible, allowing rapid antigen redesign and manufacturing in response to real-time genomic surveillance. By demonstrating complete protection with the outbreak-derived AM0059 Gc formulation, this work provides a model for how precision mRNA vaccine design can support agile outbreak preparedness for OROV and related bunyaviruses.

Recent clinical observations highlight an expanded pathogenic spectrum for OROV, including neuroinvasive disease and suspected vertical transmission leading to fetal abnormalities (5–8). Neurotropic orthobunyaviruses, including La Crosse virus and OROV, can access the CNS through hematogenous spread, with high viremia being a key determinant of neuroinvasion (5, 44, 45). Similarly, several arboviruses have demonstrated placental tropism, with viral load strongly influencing transmission risk (46–48). Because our challenge model focuses on acute systemic infection, future studies will be necessary to determine whether OROV mRNA vaccination also limits viral dissemination to neural tissues or the maternal–fetal interface. The potent neutralizing antibodies elicited by the AM0059 Gc vaccine may reduce viremia and thereby lower the probability of CNS or placental seeding. Likewise, the strong vaccine-elicited cellular immunity may help eliminate infected neural or placental cells should breakthrough infection occur. Pregnant mouse models incorporating neurotropic clinical isolates and measurement of viral burden in the brain, placenta, and fetal tissues will be essential next steps to assess whether mRNA vaccination can mitigate these increasingly recognized clinical complications.

Cumulatively, these results establish a proof-of-concept for a rapid-response, strain-adaptable mRNA vaccine platform against OROV. Demonstrating protection against both prototype and outbreak strains, this study lays a foundation for rational vaccine strategies targeting emerging and re-emerging arboviruses. The AM0059 Gc mRNA-LNP vaccine represents a strong candidate for continued preclinical development, warranting further investigation into the durability, breadth, and memory of protection across genetically distinct OROV isolates. Evaluation in translational models such as nonhuman primates will help define its immunogenic potential for human application.

Beyond OROV, these results highlight the broader utility of mRNA technology for combating arboviruses with high genetic plasticity, such as Rift Valley fever virus and Crimean-Congo hemorrhagic fever virus, for which mRNA vaccine prototypes are already under evaluation (49–52). Integrating genomic surveillance into antigen design provides a key advantage for outbreak responsiveness. Real-time adaptation to emerging variants will become increasingly vital as climate change expands the range of vector-borne pathogens. Overall, these findings validate precision mRNA vaccinology as a viable strategy for bunyaviruses and inform future efforts to develop broadly protective, rapidly deployable vaccines for emerging pathogens.

## MATERIALS AND METHODS

### Cells and virus

HEK293T (human embryonic kidney), Vero E6 (African green monkey kidney), and BHK-21 (baby hamster kidney fibroblast) cell lines were obtained from the American Type Culture Collection (ATCC). Cells were maintained in Dulbecco’s modified Eagle medium (DMEM; Gibco) supplemented with 10% fetal bovine serum (FBS; Gibco) and 1% penicillin-streptomycin (Gibco) at 37°C in a humidified incubator containing 5% CO_2_.

The recombinant prototype strain (rOROV^BeAn19991^) was kindly provided by Dr. Natasha Tilston-Lunel (Indiana University School of Medicine). The outbreak-associated strain OROV^240023^ was obtained through BEI Resources (NIAID, NIH: OROV, 240023, NR-59930).

### Virus propagation

Working stocks of rOROV^BeAn19991^ and OROV^240023^ were propagated in Vero E6 cells at a multiplicity of infection of 0.01. Infected cells were incubated at 37°C with 5% CO_2_ until the development of visible cytopathic effect. Virus-containing supernatant was harvested, clarified by centrifugation at 2,500 rpm for 10 min to remove cellular debris, aliquoted, and stored at −80°C until use.

Virus titers were determined by a plaque assay on BHK-21 cells seeded at a density of 3 × 10^5^ cells/mL in a 12-well plate. Cell monolayers were infected with 10-fold serial dilutions of the virus and incubated for 3 days at 37°C under an overlay of DMEM containing 1% methylcellulose (Sigma-Aldrich). Following incubation, cells were fixed with 4% paraformaldehyde and stained with 1% crystal violet to visualize plaques. Plaques were enumerated to calculate viral titers expressed as PFU per milliliter (PFU/mL).

### OROV vaccine construct design

Codon-optimized glycoprotein sequences encoding either the full-length GnGc polyprotein or the Gc full-length from the prototype strain BeAn19991 (GenBank accession no. KP052851.1) and the 2024 outbreak strain AM0059 (GenBank accession no. PP992528.1) were synthesized by GenScript (Piscataway, NJ, USA). The native viral signal peptide was replaced with the Lucia luciferase signal peptide (InvivoGen) to promote efficient secretion of the recombinant glycoproteins.

Each optimized coding sequence was cloned into a custom pZMV backbone containing a bacteriophage T7 promoter and 5′ and 3′ untranslated regions designed to support efficient *in vitro* transcription and translation (21). The resulting plasmids encoded either BeAn19991 GnGc, BeAn19991 Gc, AM0059 GnGc, or AM0059 Gc constructs, which were subsequently used for mRNA production and vaccine formulation.

### OROV GnGc and OROV Gc mRNA generation

*In vitro* transcription of OROV mRNA constructs was performed using the HiScribe T7 High Yield RNA Synthesis Kit (New England Biolabs, USA) with the incorporation of a CleanCap AG Cap1 analog (TriLink Biotechnologies, USA) for co-transcriptional capping. To enhance mRNA stability and minimize activation of innate immune sensors, uridine triphosphate (UTP) was completely substituted with N¹-methyl-pseudouridine triphosphate (TriLink).

Polyadenylation was carried out enzymatically at the 3′ end using *Escherichia coli* poly(A) polymerase (New England Biolabs). The resulting capped and polyadenylated mRNAs, encoding BeAn19991 or AM0059 GnGc and Gc sequences, were purified using RNA Clean & Concentrator spin columns (Zymo Research, USA) according to the manufacturer’s protocol. The integrity and polyadenylation of mRNAs were confirmed by electrophoresis on a 1% agarose gel prepared in MOPS buffer.

### DNA and mRNA transfection

HEK293T cells were transfected with 1 μg of plasmid DNA encoding either the GnGc (with or without V5-tagged) or Gc glycoproteins from the OROV BeAn19991 or AM0059 strains using PolyJet transfection reagent (SignaGen Laboratories, USA) according to the manufacturer’s instructions. For mRNA transfection, 2.5 μg of *in vitro*-transcribed OROV GnGc or Gc mRNA was introduced into HEK293T cells using the TransIT-mRNA Transfection Kit (Mirus Bio, USA).

Whole-cell lysates were collected 48 h post-transfection for DNA-transfected cells and 24 h post-transfection for mRNA-transfected cells. Total protein concentrations were quantified using the Bradford protein assay (Bio-Rad, USA) prior to subsequent analysis.

### LNP generation and transfection

mRNAs encoding either the GnGc or Gc glycoproteins from the OROV BeAn19991 and AM0059 strains were encapsulated in LNPs using GenVoy-ILM lipid (Cytiva, USA) at a mRNA-to-lipid ratio of 3:1 (vol/vol). Formulations were prepared on a NanoAssemblr Ignite+ microfluidic system (Precision NanoSystems, Canada) according to the manufacturer’s protocol. The resulting mRNA-LNPs were diluted in PBS, concentrated using Amicon Ultra centrifugal filters (Millipore, USA), and stored at 4°C until use.

mRNA concentration and encapsulation efficiency were quantified using the Quant-iT RiboGreen RNA assay (Thermo Fisher Scientific, USA). For cell transfection, 2.5 μg of mRNA-LNPs supplemented with human apolipoprotein E (Millipore Sigma, USA) in complete DMEM was added to HEK293T cells. Whole-cell lysates were collected 24 h post-transfection, and total protein concentrations were determined using the Bradford protein assay (Bio-Rad, USA).

### Western blot

Whole-cell lysates were mixed with 6× Laemmli SDS sample buffer (bioWORLD, USA), heated at 95°C for 5 min, and subjected to SDS-polyacrylamide gel electrophoresis for protein separation. Proteins were transferred onto polyvinylidene fluoride membranes using the Trans-Blot Turbo Transfer System (Bio-Rad, USA) under semi-dry conditions (25 V, 60 min).

Membranes were blocked for 1 h at room temperature in 5% (wt/vol) non-fat milk prepared in PBS containing 0.1% (vol/vol) Tween-20 (PBST), followed by overnight incubation at 4°C with a polyclonal mouse anti-OROV ascites antibody (VR-1228 AF; ATCC, USA) or anti-V5 antibody (Invitrogen, USA). After washing with PBST, membranes were incubated for 1 h at room temperature with horseradish peroxidase (HRP)-conjugated anti-mouse IgG secondary antibody (Cell Signaling Technology, USA).

For loading controls, membranes were probed with anti-GAPDH (Santa Cruz Biotechnology, USA) or anti-β-actin (Cell Signaling Technology, USA), followed by incubation with the same HRP-conjugated secondary antibody. Protein bands were visualized using the SuperSignal West Pico Chemiluminescent Substrate (Thermo Fisher Scientific, USA) and imaged with a ChemiDoc MP Imaging System (Bio-Rad, USA).

### Pairwise sequence alignment

Amino acid sequences of the OROV Gc glycoproteins from strains AM0059 (GenBank: PP992528.1) and 240023 (GenBank: PQ417949.1) were aligned using Geneious Prime (version 2024.0.1; Biomatters, Auckland, New Zealand). Alignments were generated with the Geneious Alignment algorithm configured for global alignment with free end gaps and the BLOSUM62 substitution matrix. Geneious automatically calculated sequence identity (exact residue matches) and similarity (conservative substitutions based on BLOSUM62). The final pairwise alignment was exported for figure visualization, with mismatches highlighted.

### Mice

Female BALB/c mice (6–8 weeks old; BALB/cJ, Strain# 000651; RRID: IMSR_JAX:000651) were obtained from The Jackson Laboratory (Bar Harbor, ME, USA). Female interferon-α/β receptor knockout mice (4–8 weeks old; B6.129S2-Ifnar1^tm1Agt/Mmjax^; RRID: MMRC_032045-JAX) were obtained from the Mutant Mouse Resource and Research Center at The Jackson Laboratory, an NIH-funded strain repository. This strain was originally contributed by Dr. Michael Aguet (Swiss Institute for Experimental Cancer Research) (53).

mRNA-LNP immunizations were performed under conventional housing conditions, while subsequent OROV challenge experiments were conducted in an animal biosafety level 2 facility.

### Mouse immunization

BALB/c mice were used for immunogenicity studies because they retain a fully functional type I interferon system and are widely used for profiling B cell, cytokine, and T cell responses. Hence, in this study, female BALB/c mice were immunized intramuscularly with either PBS or 3 μg of mRNA-LNPs encoding the GnGc or Gc glycoproteins from the OROV BeAn19991 or AM0059 strains. Immunizations were administered at weeks 0 and 3. Blood samples were collected at the indicated time points via retro-orbital bleeding, and sera were separated by centrifugation and heat-inactivated at 56°C for 30 min. Mice were euthanized at weeks 5 and 10, and spleens were harvested for downstream immunological analyses.

### Mouse challenge with rOROV^BeAn19991^ and OROV^240023^

A129 mice (IFNAR1^−/−^) were used exclusively for viral challenge studies because immunocompetent mouse strains do not develop clinical OROV disease. A129 mice are an established model for OROV pathogenesis and uniformly develop lethal infection, enabling rigorous assessment of vaccine-mediated protection. In this study, female A129 mice were immunized intramuscularly with either PBS or 3 μg of mRNA-LNPs encoding the Gc glycoproteins from the OROV BeAn19991 or AM0059 strains at weeks 0 and 3. At week 5, mice were challenged subcutaneously via hock injection with a lethal dose (1 × 10^4^ PFU) of rOROV^BeAn19991^ or OROV^240023^. Following challenge, animals were monitored daily for body weight, clinical signs of disease, and survival for up to 10 days post-infection.

### Enzyme-linked immunosorbent assay (ELISA)

Immulon 4 HBX 96-well plates (Thermo Fisher Scientific) were coated overnight at 4°C with purified rOROV^BeAn19991^ or OROV^240023^ virion lysate (10 µg/mL) diluted in PBS. Plates were blocked with 1% Block ACE (Bio-Rad) for 1 h at room temperature and washed three times with PBS containing 0.05% Tween-20 (PBST). Heat-inactivated mouse sera were serially diluted in PBS and incubated on the plates overnight at 4°C. After washing, bound antibodies were detected using HRP-conjugated anti-mouse IgG (Thermo Fisher Scientific). Color development was achieved using 3,3′,5,5′-tetramethylbenzidine substrate (BioLegend), and the reaction was stopped with 2 N sulfuric acid. Absorbance was measured at 450 nm using a microplate reader (BioTek). Endpoint titers were determined as the highest serum dilution yielding an absorbance value at least threefold above the background.

### Pseudovirus neutralization assay

Recombinant vesicular stomatitis virus pseudoviruses expressing OROV GnGc from either the BeAn19991 or AM0059 strain, together with a luciferase reporter gene (VSV^BeAn19991-GP^ and VSV^AM0059-GP^, respectively), were generated as previously described (21, 54, 55). Pseudoviruses were harvested 48 h post-infection, clarified by passage through 0.45 μm filters, and stored in aliquots at −80°C until use.

For neutralization assays, serial dilutions of heat-inactivated mouse sera were prepared in DMEM and incubated with pseudoviruses for 1 h at 37°C. The serum-virus mixtures were then added to HEK293T cells and incubated for 24 h under standard culture conditions. Cells were lysed, and luciferase activity was quantified as relative light units (RLU) using the Luciferase Assay System (Promega).

Percent neutralization was calculated by normalizing RLU values to control wells, where virus-only wells (no serum) represented 0% neutralization and uninfected wells (media only) represented 100%. The IC_50_ values were determined by nonlinear regression (log[serum dilution] vs normalized response, variable slope) using GraphPad Prism v.10.

### FRµNT

Neutralizing antibody titers were quantified by FRµNT. Briefly, Vero E6 cells were seeded in 96-well plates (2 × 10⁴ cells/well) and incubated overnight at 37°C to form a confluent monolayer. Mouse sera were heat-inactivated at 56°C for 30 min and serially diluted 10-fold in infection medium (DMEM supplemented with 2% FBS). Each dilution was mixed 1:1 (vol/vol) with OROV (200 PFU per well) and incubated at 37°C for 1 h to allow antibody-virus complex formation.

Following incubation, 50 µL of the serum-virus mixture was added to the Vero E6 monolayers and incubated for 1 h at 37°C with gentle rocking every 15 min. Subsequently, 100 µL of overlay medium (DMEM containing 1.5% carboxymethylcellulose and 2% FBS) was added to each well, and plates were incubated for 48 h at 37°C. Following infection, cells were fixed with ice-cold 80% acetone for 20 min at RT and air-dried.

For immunostaining, fixed monolayers were incubated with a primary mouse anti-OROV Gc antibody (Jung laboratory-generated, 1:1,000 dilution in PBS with 3% BSA) for 1 h at room temperature, followed by an HRP-conjugated anti-mouse IgG secondary antibody (1:2,000 dilution, 1 h at room temperature). Foci were visualized using the Vector VIP Substrate Kit, Peroxidase (HRP) (Vector Laboratories), according to the manufacturer’s protocol. The reaction was stopped by rinsing with distilled water, and plates were air-dried. Foci were enumerated using an ELISpot reader (CTL ImmunoSpot) with automated spot detection.

### OROV Gc-specific B cell immunophenotyping

Splenocytes (1 × 10^6^ cells) collected from immunized BALB/c mice at week 10 after initial immunization were seeded into 96-well V-bottom plate (Corning). Unstained cells were included as a negative control. Cells were incubated with TruStain FcX PLUS (BioLegend) and LIVE/DEAD Fixable Blue Dead Cell Stain (Invitrogen) in FACS buffer (2% heat-inactivated FBS in PBS) at 4°C for 15 min. After two washes with FACS buffer at 4°C for 5 min, cells were incubated with BeAn19991 Gc-Cy3 antigens along with Brilliant Stain Buffer Plus (BD Biosciences) and True-Stain Monocyte Blocker (BioLegend) in FACS buffer at 4°C for 60 min. Following wash with FACS buffer, cells were stained with OROV Gc-specific B cell immunophenotyping antibody cocktails (Table 1) in FACS buffer containing Brilliant Stain Buffer Plus and True-Stain Monocyte Blocker at 4°C for 30 min. After staining and washing, cells were fixed with 4% paraformaldehyde (PFA) at room temperature for 15 min. Prior to acquisition on the SONY ID7000 Spectral Cell Analyzer (Sony Biotechnology), cells were kept in the dark at 4°C in FACS buffer containing Tandem Stabilizer (BioLegend). The results were analyzed using FlowJo v.10.9.0 (BD Biosciences), and the gating strategy is illustrated in Fig. S4A.

**TABLE 1:** Antibody list for OROV Gc-specific B cell immunophenotyping

Antibody	Vendor	Catalog number
TruStain FcX PLUS (anti-mouse CD16/32) antibody	BioLegend	156604
Brilliant Violet 605 anti-mouse CD19 antibody	BioLegend	115539
PE/Fire 810 anti-mouse CD138 (syndecan-1) antibody	BioLegend	142545
APC anti-mouse CD93 (AA4.1, early B lineage) antibody	BioLegend	136509

### Multiplexed activation-induced marker (AIM) assay

A modified AIM assay was employed to assess OROV-specific CD4^+^ and CD8^+^ T cell responses based on previously validated protocols (56–62). Splenocytes were isolated from BALB/c mice immunized with PBS or mRNA-LNPs encoding either GnGc or Gc from the BeAn19991 or AM0059 strains at weeks 5 and 10 after initial immunization. A total of 2 × 10^6^ splenocytes per well were seeded in 96-well V-bottom plates (Corning). A CD40-blocking antibody (0.5  µg/mL) was added and incubated for 15 min to enhance specificity. Cells were then stimulated with 5  µg/mL of an overlapping 15-mer peptide pool (11-aa overlap) derived from BeAn19991 Gc (GenScript) and incubated for 20 h at 37°C. Wells containing unstimulated cells served as negative controls.

Following stimulation, cells were washed twice with FACS buffer (PBS supplemented with 2% heat-inactivated FBS and 0.5 mM EDTA), blocked with anti-CD16/CD32 (TruStain FcX PLUS; BioLegend) for 15 min, and stained for 30 min with a surface antibody cocktail in the presence of True-Stain Monocyte Blocker (BioLegend) and Brilliant Stain Buffer Plus (BD Biosciences). Cells were fixed in 4% PFA for 10 min at room temperature, washed, and resuspended in 100 µL FACS buffer containing a tandem-dye stabilizer (BioLegend). The samples were stored at 4°C in the dark until flow cytometry was performed using a SONY ID7000 Spectral Cell Analyzer (Sony Biotechnology). Data were analyzed using FlowJo software (v.10.9.0, Treestar Inc.), and the gating strategy is illustrated in Fig. S4B. A complete list of antibodies and reagents used is provided in Table 2.

**TABLE 2:** Staining cocktail and dilutions for AIM assay

Antibody	Vendor	Catalog number
Brilliant Violet 71 (anti-mouse CD11b) antibody	BioLegend	101242
Brilliant Violet 71 (anti-mouse CD19) antibody	BioLegend	115555
Brilliant Violet 785 (anti-mouse CD3) antibody	BioLegend	100232
APC-eFluor 780 (anti-mouse CD4) antibody	Thermo Fisher Scientific	47-0042-82
eFluor 450 (anti-mouse CD8a) antibody	Thermo Fisher Scientific	48-0081-82
Brilliant Violet 510 (anti-mouse CD69) antibody	BD Biosciences	563030
PerCP-eFluor 710 (anti-mouse CD137) antibody	Thermo Fisher Scientific	46-1371-82
APC (anti-mouse CD134) antibody	BioLegend	119414
FITC (anti-mouse CD40L) antibody	BioLegend	157006
Fixable Live/Dead	Thermo Fisher Scientific	L34962A
True-Stain Monocyte Blocker	BioLegend	426103
BD Horizon Brilliant Stain Buffer Plus	BD Biosciences	566385

### Olink proteomics

Splenocytes (3 × 10^5^ cells) from immunized BALB/c mice at weeks 5 and 10 following primary immunization were seeded into a 96-well V-bottom plate (Corning). Cells were stimulated *ex vivo* with 5 μg/mL of an overlapping peptide pool derived from the BeAn19991 Gc sequence, identical to that used in the AIM assay. After incubation for 24 h at 37°C, the plates were centrifuged at 400 × *g* for 7 min at 4°C, and the supernatants were transferred to 96-well clear flat-bottom Ultra-Low Attachment plates (Corning) for cytokine analysis. Cytokine levels in culture supernatants were quantified using the Olink Target 48 Mouse Cytokine panel on the Signature Q100 platform.

### Statistical analyses

All data analyses were performed using GraphPad Prism version 10 (GraphPad Software Inc.). To compare OROV-specific IgG titers and virus neutralization levels against the BeAn19991 and AM0059 strains, statistical differences were assessed using one-way ANOVA followed by unpaired *t*-tests. The results were presented as geometric means and 95% confidence intervals. Student’s *t*-test was used to evaluate the differences in CD4^+^ and CD8^+^ T cell responses measured by the AIM assay. Graphs display mean values with error bars representing the SEM. Mouse survival data were analyzed using the log-rank (Mantel-Cox) test. Statistical significance was defined as follows: *P* ≤ 0.05 (*), *P* ≤ 0.01 (**), *P* ≤ 0.001 (***), and *P* ≤ 0.0001 (****), with “ns” for non-significant findings.

## Data Availability

All the data supporting the findings of this study are available from the corresponding author upon reasonable request. The amino acid sequences of the OROV Gc glycoproteins analyzed in this study are available in Genbank under accession numbers KP052851.1 (BeAn19991), PP992528.1 (AM0059), and PQ417949.1 (OROV240023).

## SUPPLEMENTAL MATERIAL

Fig. S1 mbio.03655-25-s0001.tiff Comparison of predicted Gc structures between two Oropouche virus strains.

Fig. S2 mbio.03655-25-s0002.tiff Verification of OROV glycoprotein expression and confirmation of Gn translation using V5-tagged constructs.

Fig. S3 mbio.03655-25-s0003.tiff Percent-infection curves for authentic OROV in FRµNT using sera from vaccinated mice.

Fig. S4 mbio.03655-25-s0004.tiff Flow cytometry gating strategy for identification of OROV-specific B cells and antigen-specific T cells.

Supplemental legends mbio.03655-25-s0005.docx Legends for Fig. S1 to S4.

ASM does not own the copyrights to Supplemental Material that may be linked to, or accessed through, an article. The authors have granted ASM a non-exclusive, world-wide license to publish the Supplemental Material files. Please contact the corresponding author directly for reuse.
