# Supplementary figures and images for: Lineage-matched Oropouche virus mRNA-LNP vaccines confer complete, cross-protective immunity in mice
Source: mBio. 2026 Jan 14;17(2):e03655-25. doi: 10.1128/mbio.03655-25 (PMC12892959; doi:10.1128/mbio.03655-25)

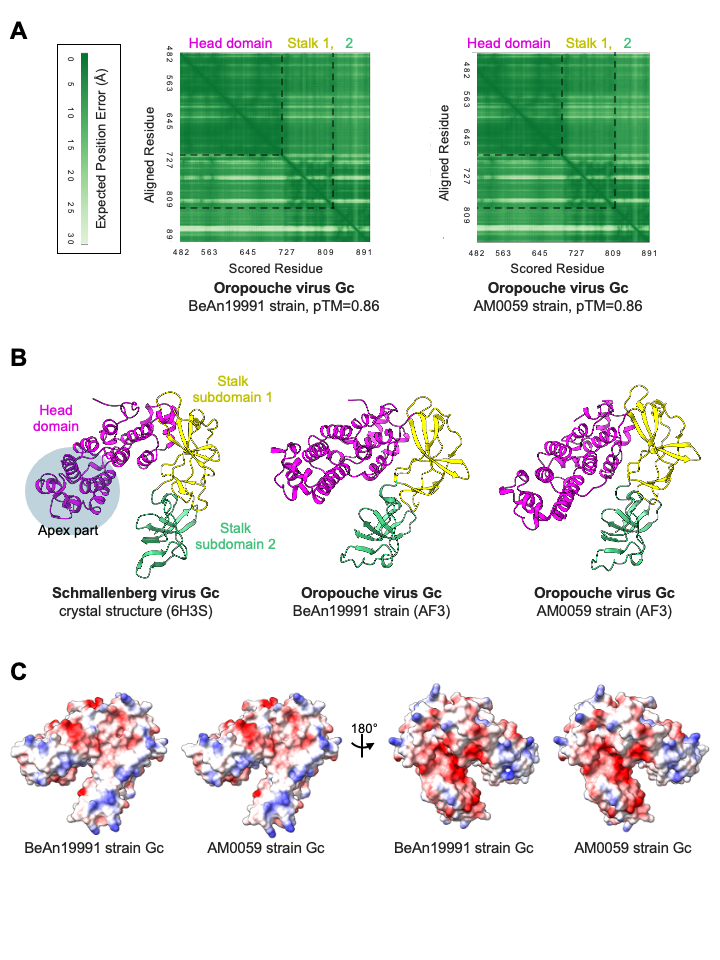

Supplement: Fig. S1 — Comparison of predicted Gc structures between two Oropouche virus strains. [file mbio.03655-25-s0001.tiff]

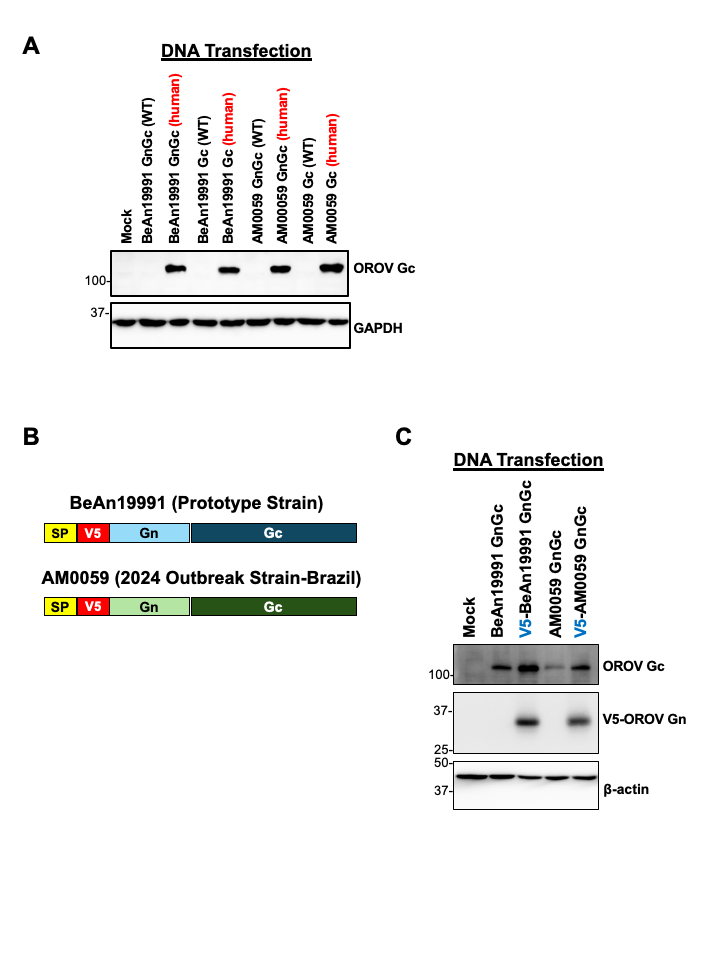

Supplement: Fig. S2 — Verification of OROV glycoprotein expression and confirmation of Gn translation using V5-tagged constructs. [file mbio.03655-25-s0002.tiff]

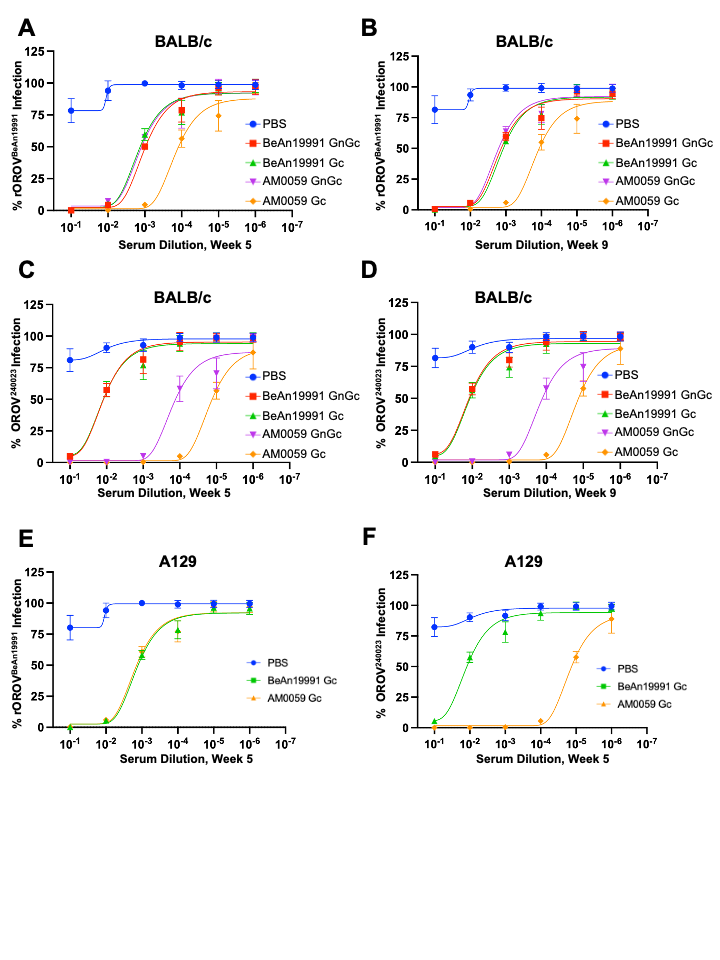

Supplement: Fig. S3 — Percent-infection curves for authentic OROV in FRµNT using sera from vaccinated mice. [file mbio.03655-25-s0003.tiff]

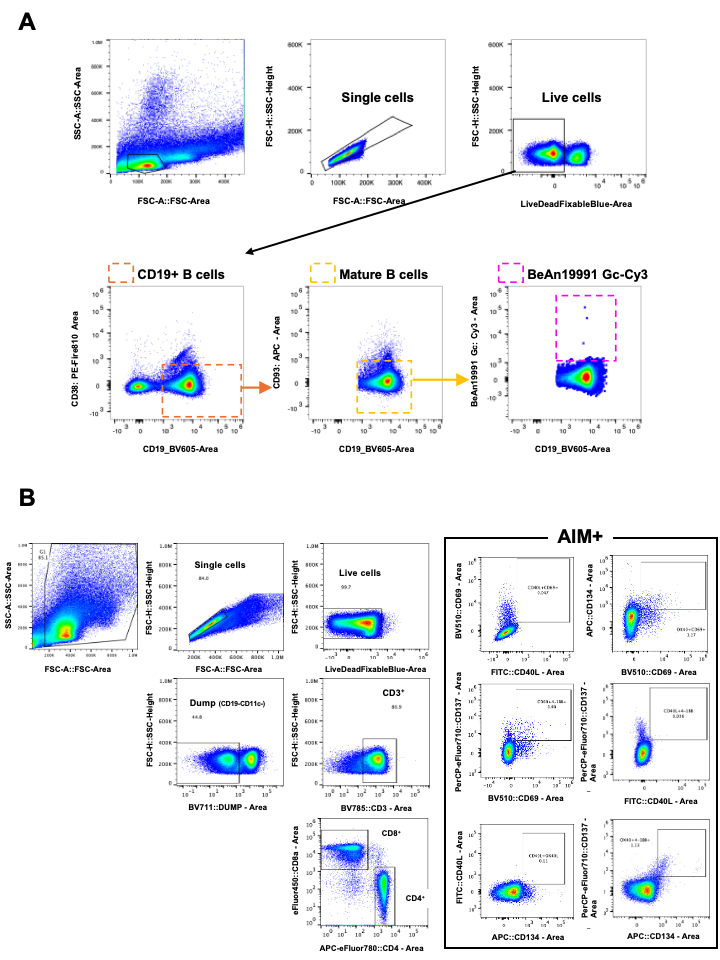

Supplement: Fig. S4 — Flow cytometry gating strategy for identification of OROV-specific B cells and antigen-specific T cells. [file mbio.03655-25-s0004.tiff]
