# Supplementary material for: Lineage-matched Oropouche virus mRNA-LNP vaccines confer complete, cross-protective immunity in mice
Source: mBio. 2026 Jan 14;17(2):e03655-25. doi: 10.1128/mbio.03655-25 (PMC12892959; doi:10.1128/mbio.03655-25)
Supplement: Supplemental legends — Legends for Fig. S1 to S4. [file mbio.03655-25-s0005.docx]

**Lineage-matched Oropouche virus mRNA-LNP vaccines confer complete,**

**cross-protective immunity in mice**

Yumiko Yamada^1,2,^*, Inho Cha^1,2,3,^*, Soowon Kang ^1,2^, Wan-Shan Yang ^1,2^, Morgan Lewis ^1,2^, Chloe Chung ^1,2^, Woo-Jin Shin ^1,2^, Jun Bae Park ^1,2^, Nam‐Hyuk Cho^4,5^, Young-Ki Choi^6^,

Natasha L Tilston ^7^, Jae U. Jung ^1,2,#^

^1^Department of Microbial Sciences in Health, Cleveland Clinic Research, Cleveland Clinic, OH, USA

^2^Global Center for Pathogen Research and Human Health Research, Cleveland Clinic Research, Cleveland Clinic, OH, USA

^3^Department of Molecular Biology and Microbiology, Case Western Reserve University School of Medicine, Cleveland, OH, USA

^4^Department of Microbiology and Immunology, Seoul National University College of Medicine, Seoul 03080, Republic of Korea

^5^Department of Biomedical Sciences, Seoul National University College of Medicine, Seoul 03080, Republic of Korea

^6^Center for Study of Emerging and Re-emerging Viruses, Korea Virus Research Institute, Institute for Basic Science, Daejeon, Republic of Korea

^7^Department of Microbiology and Immunology, Indiana University School of Medicine, Indianapolis, Indiana, USA

**Running head:** Rapid-Response OROV mRNA Vaccine

^#^Address correspondence to Jae U. Jung, [jungj@ccf.org](mailto:jungj@ccf.org)

*These authors contributed equally to this work. The author’s order was determined based on the overall contribution of the project.

**Supplementary Fig. 1. Comparison of predicted Gc structures between two Oropouche virus strains.** (A) Predicted aligned error matrices and pTM scores between BeAn19991 and AM0059 Gc generated by AlphaFold3. (B) Overall structures of the Schmallenberg virus Gc and the predicted Gc structures of OROV strains BeAn19991 and AM0059. (C) Electrostatic surface potentials of BeAn19991 strain Gc and AM0059 strain Gc.

**Supplementary Figure 2. Verification of OROV glycoprotein expression and confirmation of Gn translation using V5-tagged constructs.**

(A) Western blot analysis showing expression of OROV glycoproteins following plasmid DNA transfection in HEK293T cells. Cells were transfected with plasmids encoding either the wild-type (WT) or human codon optimized BeAn19991 or AM0059 constructs containing the full-length GnGc or Gc alone. Cell lysates were collected 24 h post-transfection and probed with anti-OROV Gc antibodies. GAPDH served as a loading control. (B) Schematic representation of V5-tagged GnGc constructs containing an N-terminal V5 epitope fused to Gn for detection. (C) Western blot analysis confirming expression of the V5-tagged GnGc constructs. β-actin was used as a loading control.

**Supplementary Fig. 3. Percent-infection curves for authentic OROV in FRµNT using sera from vaccinated mice.** Focus reduction microneutralization test (FRµNT) performed with authentic OROV strains using serially diluted sera from vaccinated (A-D) BALB/c and (E-F) A129 mice. Each panel shows percent infection of Vero E6 cells (normalized to virus-only wells) as a function of serum dilution for the vaccine groups indicated in the plot legends. Sera were collected at the stated time points (weeks 5 and/or 9) and tested against the indicated virus strains (rOROV^BeAn19991^ or OROV^240023^). Data are presented as mean ± SEM of technical replicates.

**Supplementary Fig. 4. Flow cytometry gating strategy for identification of OROV-specific B cells and antigen-specific T cells.** (A) Gating strategy for B cell analysis. Splenocytes were sequentially gated on singlets, live cells, and CD19⁺ B cells, followed by identification of mature B cells (CD19^+^CD93^-^) and BeAn19991 Gc-Cy3⁺ antigen-specific B cells. (B) Gating strategy for T cell activation analysis. Splenocytes were gated on singlets, live cells, and CD3⁺ lymphocytes after exclusion of CD19⁺CD11c⁺ dump populations. Antigen-specific activation was determined based on co-expression of CD69, 4-1BB, OX40, and CD40L following peptide restimulation (AIM⁺). Representative plots for CD4⁺ and CD8⁺ T cell are shown.
